# Multiple Dimensions of Perceived Discrimination, Race-Ethnicity, and Mortality Risk Among Older Adults

**DOI:** 10.1093/geroni/igaa057.1936

**Published:** 2020-12-16

**Authors:** Ryon Cobb

**Affiliations:** University of Georgia, North Richland Hills, Texas, United States

## Abstract

The present study utilized data from the Health and Retirement Study (N=12,988) to investigate the joint consequences of multiple dimensions of perceived discrimination on mortality risk. Perceived discrimination is based on responses from the 2006/2008 HRS waves and included everyday discrimination, the number of attributed reasons for everyday discrimination, and major lifetime discrimination. Vital status was obtained from the National Death Index and reports from key household informants (spanning 2006–2016). Cox proportional hazard models were used to estimate the risk of mortality. During the observation period, 3,494 deaths occurred. Only the number of attributed reasons for discrimination predicted mortality risk when all discrimination measures were estimated in the same model (Hazard Ratio [HR]=1.09; 95%, Confidence Interval [CI]=1.05 - 1.14), holding all else constant. Overall, the number of attributed reasons for everyday discrimination is a particularly salient risk factor for mortality in later life.

